# Guanidinium Intercalation into Layered Zirconium Phosphates and Phosphonates: Synthesis, Structure, and Ionic Conductivity

**DOI:** 10.3390/molecules31183263

**Published:** 2026-09-15

**Authors:** Klára Melánová, Tomáš Plecháček, Ludvík Beneš, Petr Kutálek

**Affiliations:** Center of Materials and Nanotechnologies, Faculty of Chemical Technology, University of Pardubice, Studentská 95, 532 10 Pardubice, Czech Republic; tomas.plechacek@upce.cz (T.P.); ludvik.benes@upce.cz (L.B.); petr.kutalek@upce.cz (P.K.)

**Keywords:** intercalation, zirconium hydrogenphosphate, zirconium phosphonate, ionic conductivity

## Abstract

Guanidinium-intercalated derivatives of α- and γ-zirconium hydrogen phosphate, zirconium 2-sulfoethylphosphonate, and zirconium 4-sulfophenylphosphonate were successfully synthesized by treatment of the host materials with aqueous guanidine carbonate solutions at room temperature. In contrast, hydrothermal treatment at 180 °C proved unsuitable for the preparation, as guanidine underwent decomposition, leading predominantly to the formation of ammonium-intercalated phases and, in some cases, partial degradation of the host structure. The synthesized materials were characterized by powder X-ray diffraction, elemental analysis, thermogravimetric analysis, and infrared spectroscopy. The combined results confirmed the incorporation of guanidinium ions into the interlayer space together with variable amounts of co-intercalated water. Spectroscopic data further indicated the formation of extensive hydrogen-bonding networks involving guanidinium ions, water molecules, and acidic groups of the host frameworks. Proton conductivity measurements revealed a pronounced dependence on relative humidity, with conductivity increasing as the water content of the materials increased. Nevertheless, the conductivities of all guanidinium intercalates remained lower than those of the corresponding parent zirconium phosphates and phosphonates. Temperature-dependent conductivity measurements suggested that proton transport proceeds predominantly via a vehicle mechanism in both hydrated and dehydrated states. The prepared intercalates exhibited reversible dehydration and rehydration behavior and retained their structural integrity during conductivity measurements.

## 1. Introduction

Zirconium phosphates and phosphonates constitute a versatile and continuously expanding class of inorganic layered materials that have attracted considerable scientific interest due to their structural diversity, rich chemistry, and broad range of potential applications [1,2,3]. Recent reviews have highlighted the growing importance of these compounds in fields such as catalysis, ion exchange, drug delivery, and proton-conducting materials for electrochemical devices [4,5]. Among their various functional properties, proton conductivity has received particular attention because of its relevance to fuel cells, sensors, and other energy-related technologies [6,7].

Three principal layered polymorphs of zirconium phosphate are known, namely the α-, γ-, and λ-modifications. Of these, α-zirconium hydrogen phosphate monohydrate, α-Zr(HPO_4_)_2_·H_2_O (**α-ZP**), exhibits the highest thermal and hydrolytic stability and has therefore been the most extensively investigated with respect to its proton transport properties. The proton conductivity of **α-ZP** is influenced by several factors, including crystal size and orientation, pellet density, temperature, and relative humidity (RH). Under room temperature and high humidity conditions (90% RH), conductivity values typically range from 10^−6^ to 10^−4^ S cm^−1^. However, a decrease in RH from 90% to 5% results in a reduction in conductivity of approximately two orders of magnitude, emphasizing the important role of water in the proton transport mechanism [7].

Considerable efforts have been devoted to modifying and improving proton transport in zirconium phosphate-based materials. Two principal strategies have emerged: (i) intercalation of basic guest molecules into the layered structure and (ii) chemical modification of the phosphate layers through the introduction of alternative acidic functional groups, leading to the formation of zirconium phosphonates. The effect of molecular intercalation on proton conductivity has been demonstrated in numerous studies. For example, propylamine-intercalated compounds, **α-ZP**·xPrNH_2_·nH_2_O, exhibited conductivity strongly dependent on both the degree of intercalation and hydration level [8]. It was found that for the same water content, the conductivity decreases with increasing *x,* whereas at a constant degree of intercalation, it increases with increasing *n*. The nature of the guest molecule also plays a crucial role. Whereas hydrazine-intercalated **α-ZP** displays conductivity several times higher than that of the parent material, intercalates containing 1,ω-alkanediamines exhibit markedly lower conductivity [9]. Similarly, anhydrous intercalates incorporating heterocyclic molecules such as pyrazole, imidazole, pyrazine, pyridazine, and pyrimidine have shown proton transport characteristics governed not only by molecular basicity but also by the orientational mobility of the guest species within the interlayer space [10,11,12].

An alternative approach involves the preparation of α-zirconium phosphonates, in which the hydroxyl group of the monohydrogen phosphate unit is replaced by an organic substituent bonded directly to phosphorus. This structural modification enables the incorporation of additional functional groups that can participate in proton transfer processes. Previous studies on zirconium carboxyalkylphosphonates [13] and 2-aminoethylphosphonate [14] have demonstrated the potential of such materials as proton conductors. Among the reported systems, the highest conductivities were achieved for mixed zirconium sulfophenylphosphonates containing hydroxymethyl [15] or ethyl groups [16] as well as for mixed zirconium sulfophenylphosphonate-phosphate materials [16,17].

In this article, we discuss the intercalation of guanidine into α- and γ-zirconium hydrogen phosphate (**α-ZP and γ-ZP**) and into zirconium 2-sulfoethylphosphonate (**ZSEtP**) and 4-sulfophenylphosphonate (**ZSPhP**), and the proton conductivity of the prepared intercalates. The effects of the guanidine precursor and synthesis conditions on the formation and composition of the intercalated phases are examined, and the structural, thermal, spectroscopic, and proton-conducting properties of the resulting materials are evaluated.

## 2. Results and Discussion

Guanidinium intercalation compounds were synthesized by reacting with the respective host materials with either guanidine carbonate or guanidine hydrochloride at room temperature or by hydrothermal treatment at 180 °C. The extent of intercalation was found to depend strongly on both the nature of the host material and the guanidine precursor employed.

**Figure 1:**
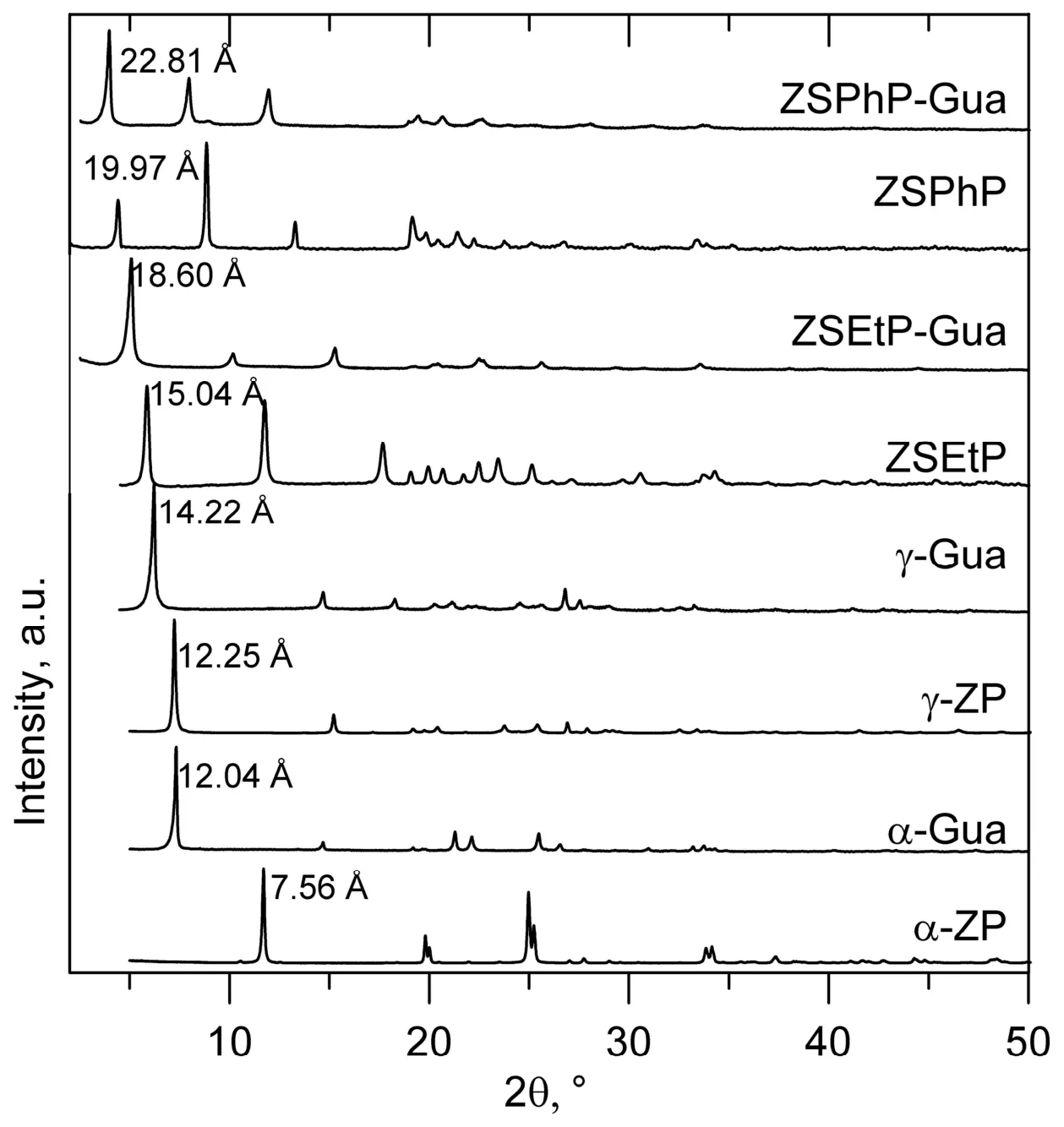
Powder X-ray diffraction patterns of the intercalates prepared from guanidine carbonate at room temperature together with diffraction patterns of corresponding hosts.

For **α-ZP**, reaction with guanidine carbonate at room temperature resulted in the formation of a single-phase intercalation compound characterized by a basal spacing of 12.04 Å (Figure 1). In contrast, treatment with guanidine hydrochloride led only to partial intercalation, as evidenced by the coexistence of reflections corresponding to both the intercalated phase and the unreacted host material (Figure S1a). A similar dependence on the guanidine precursor was observed for **γ-ZP**. The use of guanidine carbonate afforded an intercalation compound with an expanded basal spacing of 14.22 Å, whereas reaction with guanidine hydrochloride produced only a modest structural change, reflected by a decrease in basal spacing from 12.26 Å for pristine **γ-ZP** to 11.58 Å.

In contrast to the zirconium phosphates, the zirconium sulfophosphonate hosts readily underwent intercalation regardless of whether guanidine carbonate or guanidine hydrochloride was used. The resulting intercalation compounds exhibited comparable basal spacings for both guanidine sources, indicating a similar degree of guest incorporation (Figure 1 and Figure S1c,d).

The observed differences in guanidine intercalation from chloride and carbonate salts are likely related to the acidity of the host materials. Zirconium hydrogen phosphates, which contain comparatively less acidic phosphate groups, facilitate guanidinium intercalation only when guanidine is supplied as a salt of a weak acid, such as guanidinium carbonate. In contrast, sulfophenylphosphonates possessing strongly acidic sulfonic acid groups within the interlayer region readily accommodate guanidinium ions from both guanidinium chloride and guanidinium carbonate.

Hydrothermal treatment at 180 °C was found to be unsuitable for the synthesis of guanidinium intercalates. The products obtained under these conditions exhibited significantly smaller basal spacings than the corresponding materials prepared at room temperature (Figure S2), suggesting a lower degree of intercalation. Furthermore, hydrothermal reactions of sulfophosphonates with guanidine carbonate resulted in a substantial loss of crystallinity, yielding nearly amorphous products. This observation indicates that partial decomposition of the layered host structure likely occurred during the hydrothermal treatment (Figure S2g,h).

The compositions of the prepared intercalation compounds were determined by a combination of elemental analysis and thermogravimetric measurements. The calculated compositions and corresponding chemical formulas are summarized in Table S1. Intercalates synthesized at room temperature using guanidine carbonate were found to contain approximately one guanidinium ion per formula unit together with varying amounts of interlayer water. Similar compositions were obtained for the guanidinium-intercalated derivatives of **ZSEtP** and **ZSPhP** prepared from guanidine hydrochloride. Energy-dispersive X-ray spectroscopy (EDX) confirmed that the composition of the host layers remained unchanged after treatment with guanidinium carbonate at room temperature. For the zirconium phosphate intercalates, the P/Zr molar ratio was determined to be 2.0 ± 0.1, identical to that of the parent host material. Similarly, the elemental composition of the intercalated sulfophenylphosphonates was consistent with that of the corresponding hosts. The P/Zr ratio remained close to the theoretical value 2, while the S/Zr ratio was approximately 2.0 ± 0.1 for ZSEtP and 1.8 ± 0.1 for ZSPhP.

The products obtained under hydrothermal conditions at 180 °C exhibited markedly different compositions. For **α-ZP**, elemental analysis revealed only trace amounts of carbon, indicating extensive decomposition of guanidine during the reaction and the preferential formation of ammonium-intercalated phases. A similar outcome was observed for **γ-ZP** treated with guanidine carbonate, where the product corresponded to an ammonium intercalate. In contrast, the reaction of **γ-ZP** with guanidine hydrochloride afforded a guanidinium intercalate containing approximately 0.5 guanidinium species per formula unit. Hydrothermal treatment of sulfophosphonates with guanidine hydrochloride resulted in the formation of an ammonium-intercalated **ZSEtP** [18] and a two-phase product in the case of **ZSPhP**. The materials obtained from reactions with guanidine carbonate were nearly amorphous, and therefore their composition was not analyzed.

The combined results of powder X-ray diffraction and elemental analysis demonstrate that intercalation using guanidine carbonate at room temperature provides the most effective route to the formation of well-defined guanidinium intercalation compounds. Consequently, only samples prepared under these conditions were selected for further structural and physicochemical characterization. The compositions of the investigated intercalates and the abbreviations used throughout this work are summarized in Table 1; their schematic representations are shown in Scheme S1.

**Figure 2:**
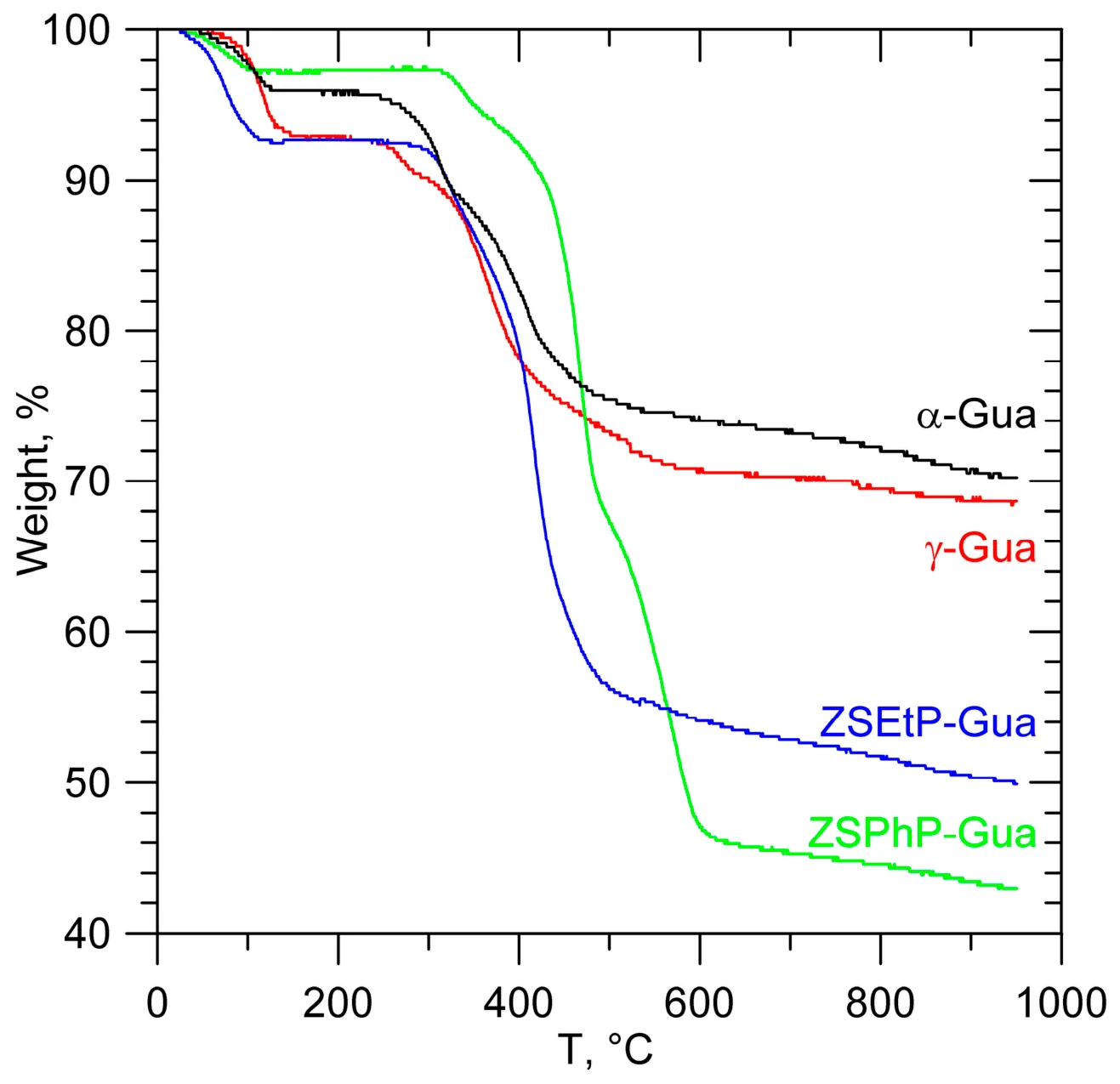
Thermogravimetric curves of intercalates prepared from guanidinium carbonate at room temperature.

All four prepared intercalation compounds contained a certain amount of interlayer water, as confirmed by thermogravimetric analysis. Thermogravimetric curves of the four guanidinium intercalates prepared using guanidine carbonate are shown in Figure 2. In all cases, the thermal decomposition proceeds in two distinct steps. The first mass-loss step, occurring below approximately 110–150 °C, corresponds to the release of interlayer water molecules. Following dehydration, the anhydrous phosphate intercalates **α-Gua** and **γ-Gua**, remained thermally stable up to approximately 240 °C. At higher temperatures, the decomposition of the intercalated guanidinium species takes place simultaneously with the condensation of hydrogen phosphate groups. In contrast, the guanidinium-intercalated sulfophosphonates, **ZSEtP-Gua** and **ZSPhP-Gua**, exhibited enhanced thermal stability. In these materials, decomposition of the intercalated guanidinium species, accompanied by degradation of the phosphonate framework, commences only above 300 °C. Thus, the sulfophosphonate hosts provide a more thermally stable environment for the incorporated guanidinium ions than the corresponding zirconium phosphate hosts. The end products of thermal decomposition of all four compounds are ZrP_2_O_7_ (JCPDS No. 04-009-1102) [19].

SEM images of the intercalates prepared from guanidine carbonate at room temperature are shown in Figure S3. No significant morphological differences are observed among the samples investigated. All intercalates consist of agglomerates of thin platelet-like particles, indicating that the intercalation process does not substantially alter the overall morphology of the parent layered materials.

**Figure 3:**
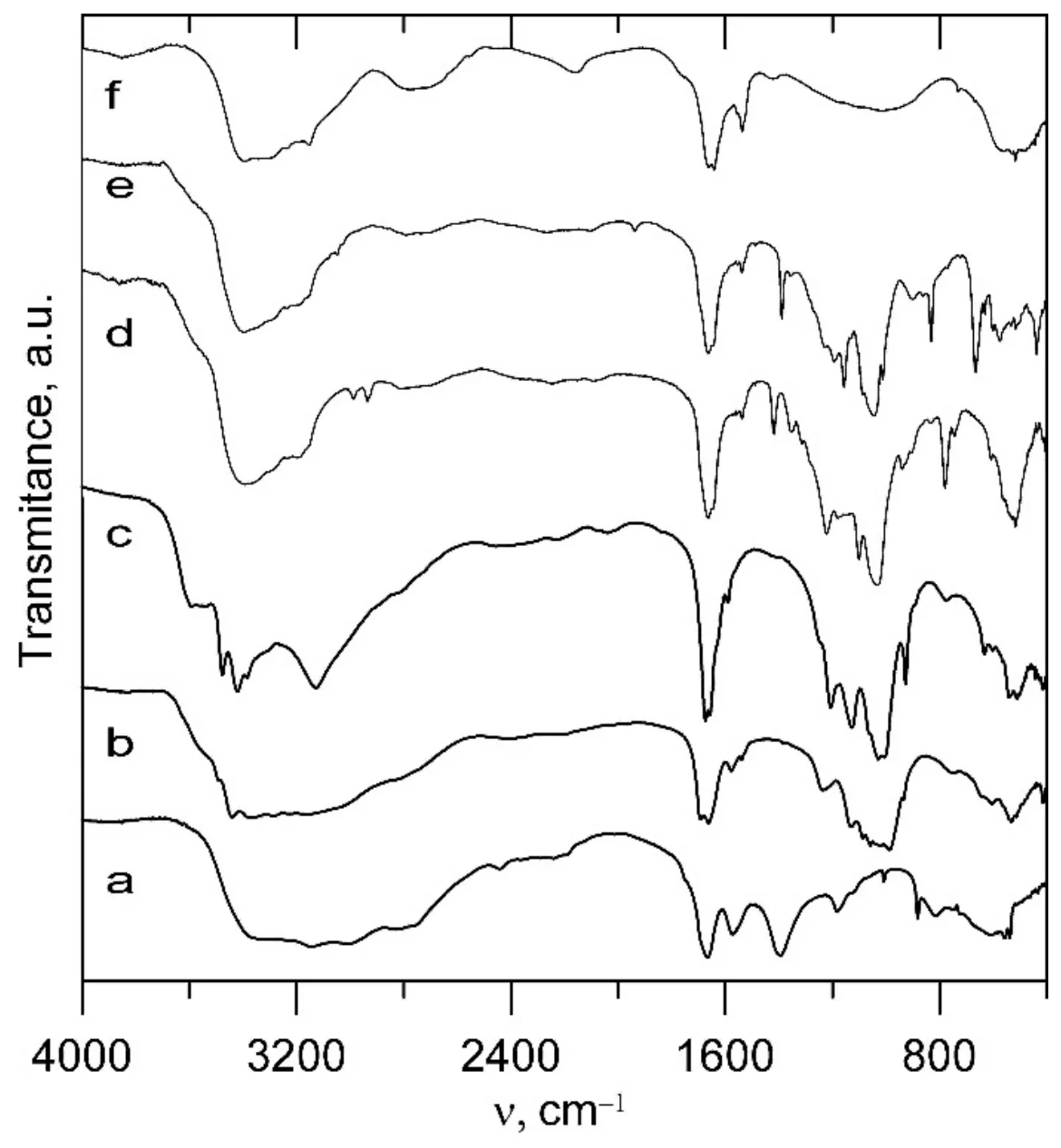
Infrared spectra of guanidine carbonate (a), **α-Gua** (b), **γ-Gua** (c), **ZSEtP-Gua** (d), and **ZSPhP-Gua** (e) and guanidine hydrochloride (f).

The FTIR spectra of all prepared intercalation compounds confirm the presence of guanidinium species within the interlayer space (Figure 3). Characteristic absorption bands assigned to the degenerate ν(CN_3_) stretching and δ(NH_2_) scissoring vibrations of the guanidinium cation are observed in all spectra [20]. For the zirconium phosphate intercalates, these bands appear at 1662 and 1577 cm^−1^ for **α-Gua** and at 1657 and 1590 cm^−1^ for **γ-Gua**. These frequencies are very close to those reported for guanidine carbonate (1665 and 1572 cm^−1^), suggesting that the vibrational environment of the guanidinium ions in the zirconium phosphate intercalates is broadly similar to that in guanidine carbonate.

In the sulfophosphonate-based intercalates, the guanidinium bands are shifted to lower wavenumbers. For **ZSPhP-Gua**, absorptions are observed at 1647 and 1538 cm^−1^, values that closely correspond to those of guanidine hydrochloride (1641 and 1537 cm^−1^). Likewise, **ZSEtP-Gua** exhibits characteristic guanidinium bands at 1665 and 1537 cm^−1^. The observed variations in band positions reflect differences in the hydrogen-bonding interactions and local environments experienced by the guanidinium ions in the various host structures.

All spectra also exhibit an intense broad absorption centered near 1000 cm^−1^, which can be attributed to stretching vibrations of phosphate and phosphonate groups constituting the host layers. Furthermore, a broad band extending across the 3200–3600 cm^−1^ region is present in all samples, indicating extensive hydrogen bonding involving intercalated guanidinium cations, water molecules, and oxygen-containing groups of the host layers. These results are consistent with the proposed compositions of the intercalation compounds and support the formation of hydrogen-bonded supramolecular networks within the interlayer galleries.

The proton conductivities of the guanidinium-intercalated materials were evaluated at room temperature under controlled relative humidities of 25%, 40%, and 75%. Representative complex impedance plots recorded in the frequency range from 0.1 Hz to 10^6^ Hz are shown in Figure S4. All samples exhibited impedance responses characteristic of ionic conductors, consisting of a semicircle in the high-frequency region with a linear tail in the low-frequency region. The conductivity values were calculated from the impedance data according to the procedure described in the Experimental section, and the resulting values are summarized in Table 2.

For all investigated intercalates, the conductivity increased with increasing relative humidity, indicating that proton transport is strongly influenced by the presence of adsorbed or interlayer water. This behavior is typical of layered zirconium phosphates and phosphonates, where water molecules facilitate proton migration through hydrogen-bonded networks. Among the studied materials, **γ-Gua** exhibited the lowest conductivity over the entire humidity range, whereas **ZSPhP-Gua** showed the highest conductivity. The superior performance of **ZSPhP-Gua** can be attributed to the presence of strongly acidic sulfonic groups and the resulting extensive hydrogen-bonding network, which provides favorable pathways for proton transport.

Direct comparison of the obtained conductivity values with literature data is complicated by differences in measurement conditions, particularly relative humidity and temperature. Nevertheless, several trends can be identified. The conductivity of **ZSEtP-Gua** was substantially lower than that reported for the parent zirconium sulfoethylphosphonate, for which a conductivity of 1.6 × 10^−3^ S cm^−1^ was measured at room temperature and 75% RH [21]. Similarly, the conductivities of the phosphate-based intercalates were lower than those reported for the corresponding host materials. Crystalline α-Zr(HPO_4_)_2_·H_2_O exhibits conductivities in the range of 10^−6^ to 10^−4^ S cm^−1^ at room temperature and 90% RH [7,22], while γ-zirconium phosphate typically shows conductivities between 10^−6^ and 10^−5^ S cm^−1^ depending on humidity [23]. Likewise, the conductivity of **ZSPhP-Gua** remained below the value of approximately 10^−3^ S cm^−1^ reported for the parent sulfophenylphosphonate material at 70 °C and 75% RH [17]. The conductivity values of our intercalates are clearly lower than those of zirconium fluorophosphates—layered (NH_4_)_2_[ZrF_2_(HPO_4_)_2_] [24], three-dimensional (NH_4_)_5_[Zr_3_(OH)_3_F_6_(PO_4_)_2_(HPO_4_)] [25] and one-dimensional (NH_4_)_2_[ZrF(PO_4_)(HPO)_4_] [26], which range from 10^−6^ to 10^−2^ S cm^−1^ depending on temperature and RH.

Our results indicate that incorporation of guanidinium ions into the interlayer space does not enhance proton transport in the investigated zirconium phosphate and phosphonate hosts. On the contrary, partial replacement of mobile protons by guanidinium cations and modification of the hydrogen-bonding network appear to reduce the overall proton conductivity relative to the corresponding parent materials.

**Figure 4:**
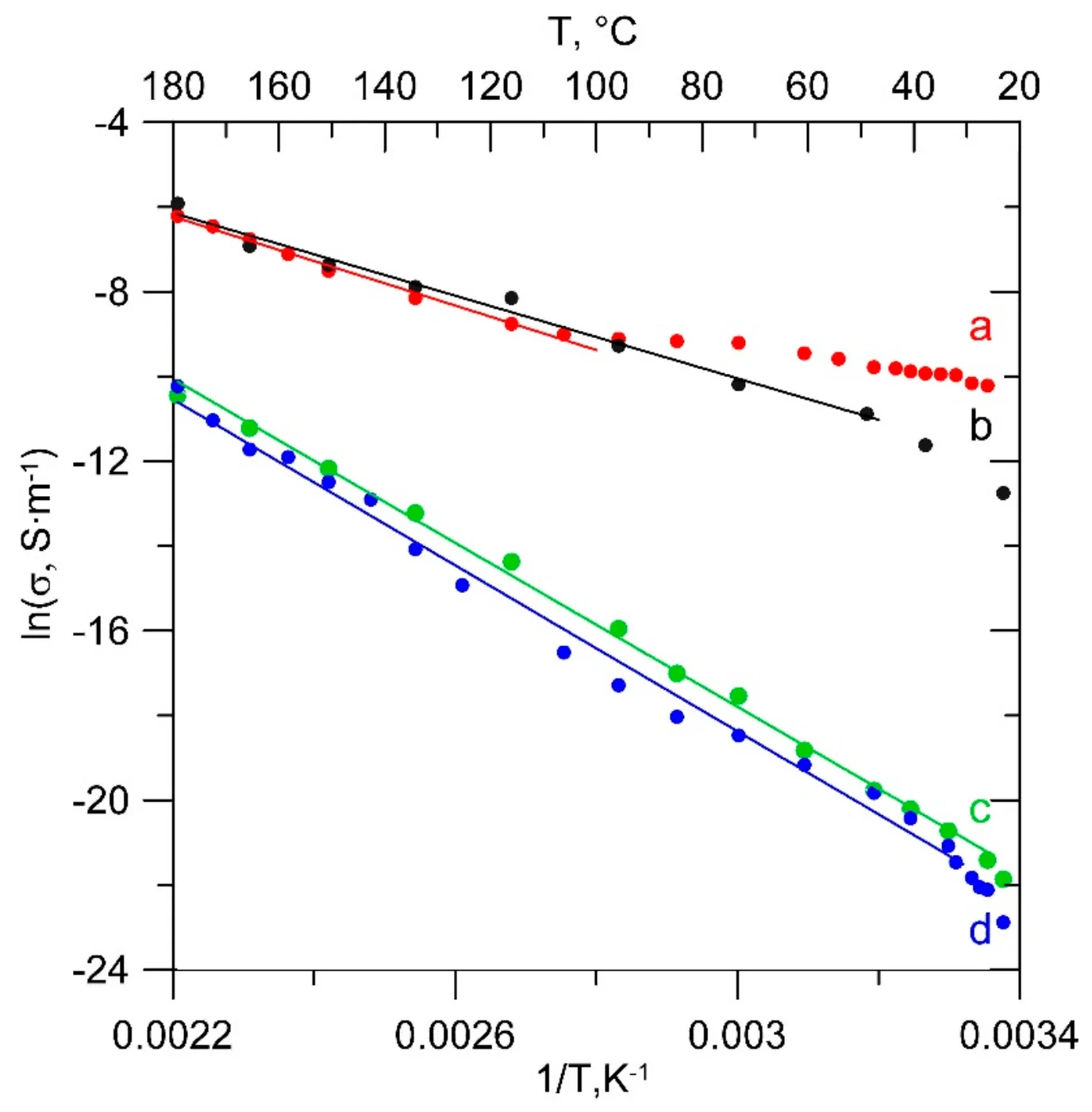
Arrhenius plots for **ZSPhP-Gua** (a), **ZSEtP-Gua** (b) at 25% RH and dehydrated **ZSEtP-Gua** (c) and dehydrated **ZSPhP-Gua** (d) at 0% RH.

The temperature dependence of proton conductivity was investigated for **ZSEtP-Gua** and **ZSPhP-Gua** under 25% of relative humidity from room temperature up to 180 °C. The corresponding Arrhenius plots are presented in Figure 4a,b, selected complex impedance plots are shown in Figure S5. Both materials exhibit changes in conductivity that correlate with their thermal dehydration and structural evolution.

**Figure 5:**
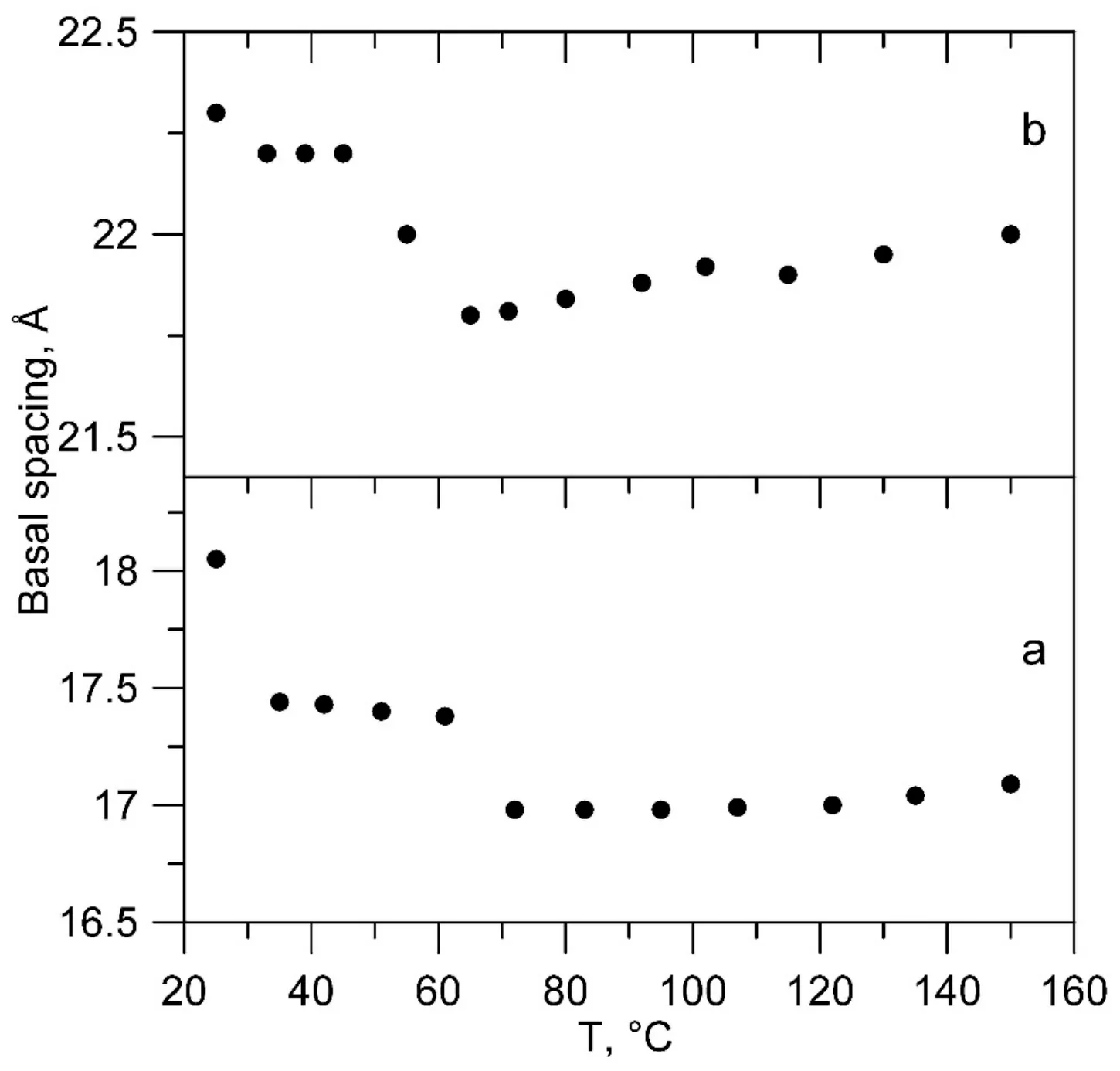
The dependence of basal spacing of **ZSEtP-Gua** (a), **ZSPhP-Gua** (b) on temperature.

For **ZSEtP-Gua**, the dependence of lnσ on 1/T can be divided into two distinct regions. At lower temperatures, proton transport is likely facilitated by the presence of co-intercalated water molecules, which participate in an extended hydrogen-bonding network within the interlayer space. Upon heating, gradual dehydration occurs, accompanied by a decrease in the basal spacing, as evidenced by the temperature-dependent X-ray diffraction data (Figure 5). Above approximately 50 °C, a linear Arrhenius relationship is observed, indicating a change in the proton transport mechanism. In this temperature range, where most interlayer water has been removed, proton conduction is presumed to occur predominantly through pathways involving the guanidinium ions and the host framework. A similar behavior was observed for **ZSPhP-Gua**. However, dehydration proceeds over a broader temperature interval and is completed only at temperatures close to 100 °C.

The activation energies determined from the linear high-temperature regions of the Arrhenius plots are 0.41 eV for **ZSEtP-Gua** and 0.47 eV for **ZSPhP-Gua**. These values are consistent with proton transport governed predominantly by a vehicle mechanism [27,28], in which protonated species migrate through the structure rather than proton transfer occurring exclusively by structural diffusion along a hydrogen-bonded network.

The temperature dependence of proton conductivity was also investigated for pre-dehydrated samples under anhydrous conditions (0% RH), and the corresponding Arrhenius plots are shown in Figure 4c,d, for complex impedance plots, see Figure S6. As expected, removal of the co-intercalated water resulted in a substantial decrease in conductivity at room temperature, highlighting the important contribution of water molecules to proton transport in the hydrated materials. Nevertheless, the conductivity of both compounds increased steadily with increasing temperature. At 180 °C, maximum conductivity values of 3.6 × 10^−7^ S cm^−1^ and 2.9 × 10^−7^ S cm^−1^ were obtained for **ZSEtP-Gua** and **ZSPhP-Gua**, respectively.

These conductivities are comparable to, or slightly higher than, those reported for anhydrous zirconium phosphates at the same temperature (8 × 10^−8^ S cm^−1^ for both **α-ZP** and **γ-ZP** [22,23]). However, they remain significantly lower than the conductivities observed for α-ZP intercalated with hydrazine [9] or imidazole [10], or zirconium phosphates (NH_4_)_2_[ZrF_2_(HPO_4_)_2_] (1.1 × 10^−5^ S cm^−1^ at 230 °C) [24], (NH_4_)Zr(H_0.66_PO_4_)_3_ (1.45 × 10^−3^ S cm^−1^ at 180 °C) [29], or ZrH_5_(PO_4_)_3_ (0.5–3.11 × 10^−2^ S cm^−1^) [30]. The activation energies derived from the Arrhenius plots were identical for both materials, with a value of 0.84 eV. Such relatively high activation energies suggest that proton transport proceeds predominantly via a vehicle mechanism involving the migration of protonated species rather than by a Grotthuss-type hopping process.

To assess the structural stability of the intercalates during conductivity measurements, powder X-ray diffraction and FTIR spectra were recorded after the heating cycle and subsequent rehydration. As shown in Figure S7, no significant differences were observed between the diffraction patterns or infrared spectra obtained before and after the conductivity experiments. These results demonstrate that the dehydration-rehydration process is reversible and confirm the good structural stability of both **ZSEtP-Gua** and **ZSPhP-Gua** under the applied measurement conditions.

## 3. Materials and Methods

### 3.1. Syntheses

All commercially available chemicals were obtained from the commercial provider Merck s.r.o., Prague, Czech Republic, Host compounds, α-Zr(HPO_4_)_2_·H_2_O (**α-ZP**) [31], γ-Zr(H_2_PO_4_)(PO_4_)·2H_2_O (**γ-ZP**) [32], Zr(HO_3_SC_2_H_4_PO_3_)_2_·2H_2_O (**ZSEtP**) [21], and zirconium sulfophenylphosphonate-phenylphosphonate Zr(HO_3_SC_6_H_4_PO_3_)_1,8_(C_6_H_5_PO_3_)_0.2_·2H_2_O (**ZSPhP**) [17] were prepared according to the previously described procedures.

Guanidine-intercalated derivatives were prepared by dispersing the host material (1 mmol) in an aqueous solution (50 mL) containing guanidine hydrochloride or guanidine carbonate (5 mmol). The suspensions were stirred at room temperature for 3 days to allow intercalation of guanidine into the layered structures.

Hydrothermal syntheses were carried out by suspending the host compound (0.5 mmol) in an aqueous solution (9 mL) of guanidine hydrochloride or guanidine carbonate (2.5 mmol). The reaction mixture was transferred into a 23 mL Teflon-lined Parr acid digestion bomb and heated under autogenous pressure at 180 °C for 24 h.

After synthesis, the solid products were separated by centrifugation, washed three times with deionized water, and dried in air at room temperature.

### 3.2. Characterization

**Elemental Analysis.** The chemical compositions of the prepared materials were determined by a combination of energy-dispersive X-ray (EDX) spectroscopy and organic elemental analysis. EDX measurements to check molar ratios of P/Zr and S/Zr were performed using a JEOL JSM-5500LV scanning electron microscope (Jamagata, Yamaguchi, Japan) equipped with an IXRF Systems energy-dispersive X-ray microanalyzer (IXRF Systems, Inc., Austin, TX, USA) fitted with a GRESHAM Sirius 10 detector Gresham Scientific Instruments Ltd., High Wycombe, UK). The accelerating voltage was set to 20 kV. Carbon, hydrogen, nitrogen, and sulfur contents were determined by conventional organic elemental analysis using a Flash 2000 CHNS Elemental Analyzer, (Thermo Fisher Scientific, Milano, Italy).

**Thermogravimetric Analysis.** Thermogravimetric analysis (TGA) was performed using a custom-built apparatus consisting of a computer-controlled furnace coupled with a Sartorius BP210 S balance (Sartorius, Göttingen, Germany). Measurements were conducted in air over the temperature range of 30–960 °C at a heating rate of 5 °C min^−1^.

**Powder X-ray Diffraction.** Powder X-ray diffraction (PXRD) patterns were collected using a D8 ADVANCE DAVINCI diffractometer (Bruker AXS, Karlsruhe, Germany) equipped with a Bragg–Brentano θ-θ goniometer (radius 280 mm) and a LynxEye XE-T detector. Cu Kα radiation (λ = 1.5418 Å) was employed, with the X-ray generator operated at 40 kV and 30 mA. Data were recorded at room temperature over the 2θ range of 2–50° using a step size of 0.01° and a counting time of 1 s per step (total step time 192 s). For powder X-ray diffraction measurements at higher temperatures, the sample was placed on a heated copper plate and the powder diffraction pattern in the range of 3–26° (2θ) was measured 15 min after reaching the desired temperature.

**Infrared Spectroscopy.** Fourier-transform infrared (FTIR) spectra were collected in the 400–4000 cm^−1^ range using a Thermo Nicolet NEXUS 870 FTIR (Madison, WI, USA) spectrometer equipped with a DTGS TEC detector. Spectra were recorded at a resolution of 2 cm^−1^ using 64 scans per sample. Powdered samples were analyzed ex situ in transmission mode as KBr pellets. All spectra were corrected for atmospheric water vapor and carbon dioxide contributions.

**Proton Conductivity Measurements.** Samples for impedance spectroscopy were prepared by pressing approximately 0.1 g of powdered material into rectangular pellets with a thickness (*L*) of approximately 0.3 cm and an electrode area (*A*) of approximately 0.15 cm^2^. Electrical contacts were formed using conductive carbon paste (Conductive Carbon Paste after Göcke, Neubauer Chemikalien, Münster, Germany). Impedance spectroscopy measurements were performed using a Metrohm Autolab PGStat12 system (ECO CHEMIE, Utrecht, The Netherlands) over the frequency range 0.1 Hz to 10^6^ Hz with an AC amplitude of 100 mV. The impedance spectra were analyzed using an equivalent-circuit approach implemented in ZSimpWin software, version 3.50 [33]. The sample resistance *R* obtained from the fitting procedure was used to calculate the proton conductivity *σ* according to the relation σ = *L*/(*AR*), where *L* is the pellet thickness and *A* is the electrode area. Measurements under controlled relative humidity were conducted in a sealed cell containing saturated salt solutions to establish the desired humidity conditions [34]. Conductivity measurements of dehydrated intercalates were carried out in a flow of dry nitrogen.

## 4. Conclusions

Guanidinium intercalation compounds of α- and γ-zirconium phosphate as well as zirconium 2-sulfoethylphosphonate and 4-sulfophenylphosphonate were successfully prepared by reaction with guanidine carbonate under ambient conditions. Structural characterization confirmed the incorporation of guanidinium ions into the interlayer space of all host materials. Hydrothermal synthesis at 180 °C was found to be unsuitable due to guanidine decomposition and, in some cases, partial degradation of the host structure. Thermal and spectroscopic analyses further verified the composition of the intercalates and revealed the presence of hydrogen-bonded networks involving guanidinium ions, interlayer water molecules, and the host layers.

The proton conductivity of all four intercalates increased with increasing relative humidity, demonstrating the important role of water in proton transport. However, the measured conductivities were generally lower than those of the corresponding parent zirconium phosphates and phosphonates, indicating that guanidinium incorporation does not enhance proton conduction in these systems. Temperature-dependent conductivity measurements revealed a transition from water-assisted proton transport in the hydrated materials to a less efficient conduction process in the dehydrated state. The activation energies obtained for both hydrated and anhydrous samples suggest that proton transport proceeds predominantly via a vehicle mechanism. Despite the moderate conductivity values, the sulfophosphonate intercalates exhibited good thermal and structural stability, with fully reversible dehydration and rehydration behavior.

## Figures and Tables

**Table 1:** Designation and composition of the intercalates prepared from guanidine carbonate at room temperature.

Sample	Host	Formula
**α-Gua**	**α-ZP**	Zr(HPO_4_)_2_·1.25(CH_5_N_3_)·H_2_O
**γ-Gua**	**γ-ZP**	Zr(H_2_PO_4_)(PO_4_)·0.9(CH_5_N_3_)·H_2_O
**ZSEtP-Gua**	**ZSEtP**	Zr(HO_3_SC_2_H_4_PO_3_)_2_·0.85(CH_5_N_3_)·H_2_O
**ZSPhP-Gua**	**ZSPhP**	Zr(HO_3_SC_6_H_4_PO_3_)_1.8_(C_6_H_5_PO_3_)_0.2_·0.95(CH_5_N_3_)·H_2_O

**Table 2:** Proton conductivity of the guanidinium-intercalated materials measured at room temperature at 25%, 40%, and 75% RH.

Sample	Conductivity S cm^−1^		
	25% RH	40% RH	75% RH
**α-Gua**	1.69 × 10^−8^	5.73 × 10^−8^	8.80 × 10^−7^
**γ-Gua**	8.64 × 10^−9^	1.43 × 10^−8^	4.45 × 10^−7^
**ZSEtP-Gua**	1.71 × 10^−8^	1.77 × 10^−8^	1.46 × 10^−5^
**ZSPhP-Gua**	1.55 × 10^−7^	7.00 × 10^−7^	1.51 × 10^−6^

## Data Availability

The original contributions presented in this study are included in the article/Supplementary Materials.

## Supplementary Materials

The following supporting information can be downloaded at: https://www.mdpi.com/article/10.3390/molecules31183263/s1, Figure S1: Powder X-ray patterns of guanidine intercalates prepared from guanidine hydrochloride at room temperature; Figure S2: Powder X-ray patterns of intercalates prepared at 180 °C; Figure S3: SEM images of **α-ZP**, **γ-ZP**, **ZSEtP-Gua** and **ZSPhP-Gua**; Figure S4: Complex impedance plots of the intercalates at 25 °C with variation in RH; Figure S5: Complex impedance plots of **ZSEtP-Gua** and **ZSPhP-Gua** at various temperatures.; Figure S6: Complex impedance plots of dehydrated **ZSEtP-Gua** and **ZSPhP-Gua** at various temperatures; Figure S7: Powder X-ray diffraction pattern of **ZSEtP-Gua** and **ZSPhP-Gua and** their IR spectra after conductivity measurement and rehydration; Table S1: Formulas of layered phosphonates prepared together with results of EDX, elemental and thermogravimetric analyses. Scheme S1: Schematical representation of the intercalates **α-ZP**, **γ-ZP**, **ZSEtP-Gua** and **ZSPhP-Gua**.
