# Supplementary material for: Guanidinium Intercalation into Layered Zirconium Phosphates and Phosphonates: Synthesis, Structure, and Ionic Conductivity
Source: Molecules. 2026 Sep 15;31(18):3263. doi: 10.3390/molecules31183263 (PMC13609002; doi:10.3390/molecules31183263)
Supplement: Supplementary file 1 [file molecules-31-03263-s001.zip › molecules-4524449-supplementary.pdf]

Guanidinium Intercalation into Layered Zirconium Phosphates and Phosphonates: Synthesis, Structure, and Ionic Conductivity

Klára Melánová*, Tomáš Plecháček, Ludvík Beneš and Petr Kutálek

Center of Materials and Nanotechnologies, Faculty of Chemical Technology University of Pardubice, Studentská 95, 532 10 Pardubice, Czech Republic; klara.melanova@upce.cz, tomas.plechacek@upce.cz, ludvik.benes@upce.cz, petr.kutalek@upce.cz

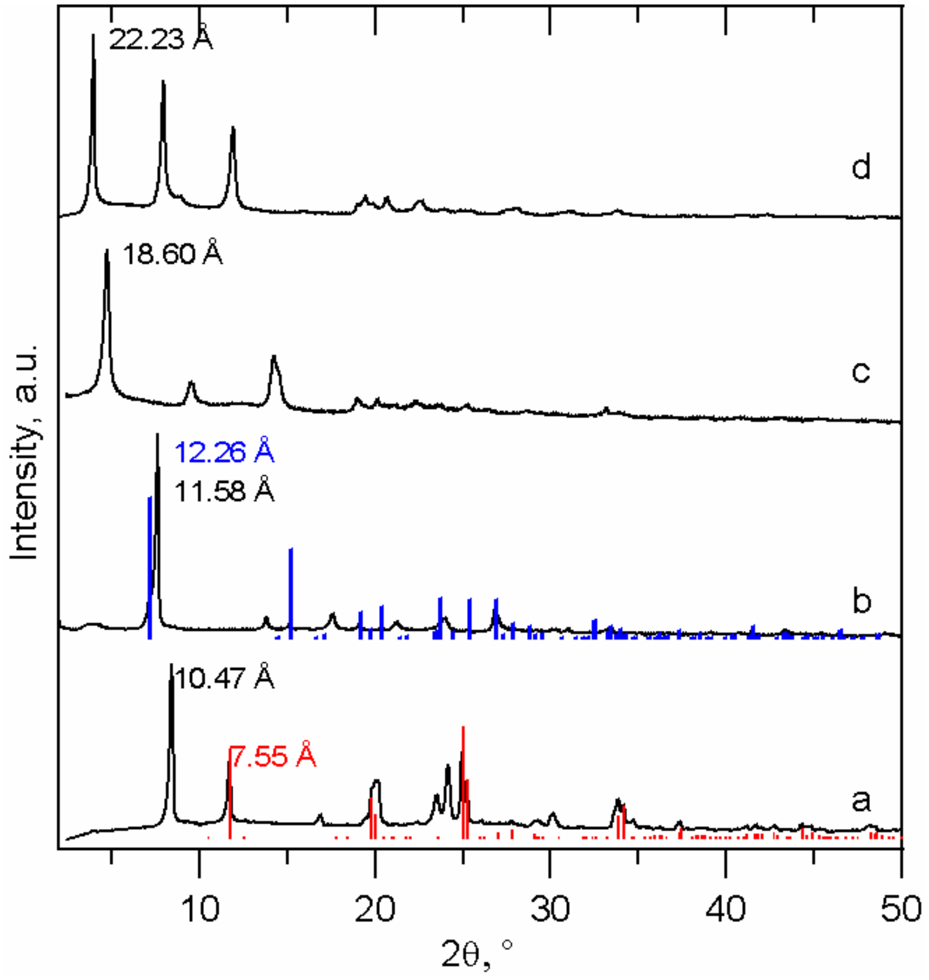

Figure S1. Powder X-ray patterns of guanidine intercalates of **α-ZP** (a), **γ-ZP** (b), **ZSEtP** (c), and **ZSPhP** (d) prepared from guanidine hydrochloride at room temperature. The blue lines correspond to **γ-ZP** (JCPDS No. 04-012-1442) and the red ones to α-ZP (JCPDS No. 04-010-6268).

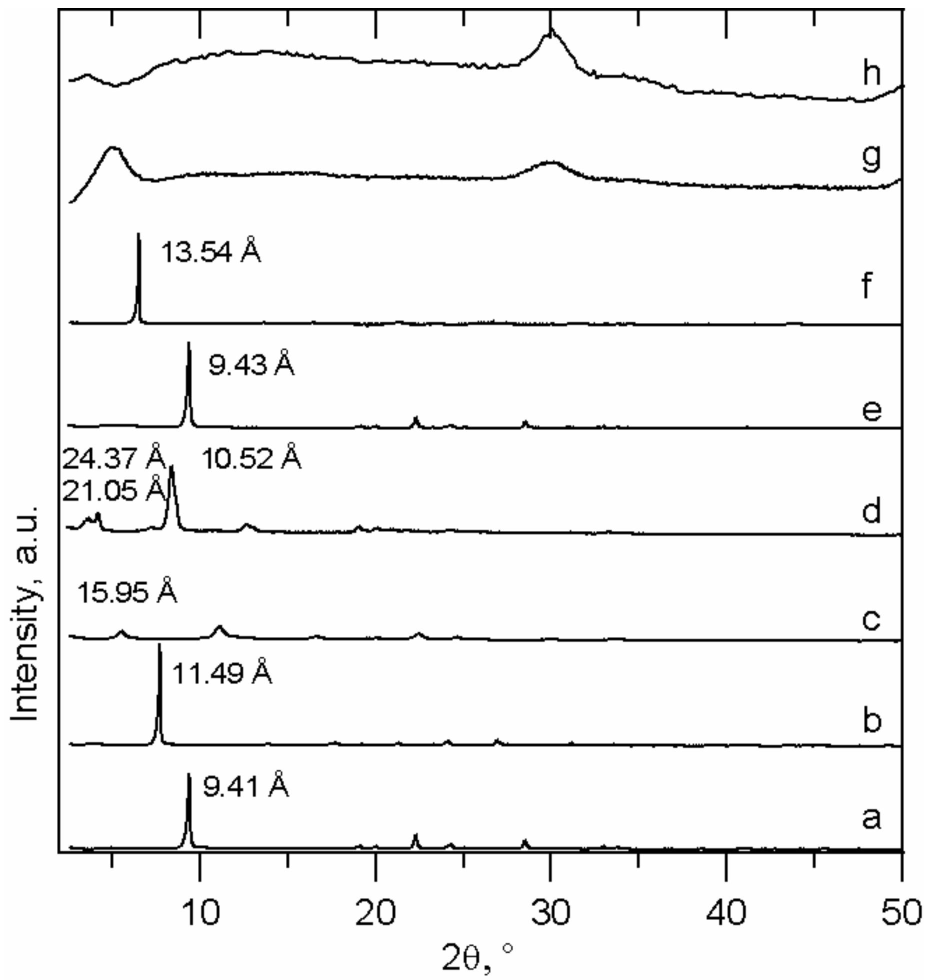

Figure S2. Powder X-ray patterns of intercalates prepared from guanidine hydrochloride and **α-ZP** (a), **γ-ZP** (b), **ZSEtP** (c), and **ZSPhP** (d) and guanidine carbonate with **α-ZP** (e), **γ-ZP** (f), **ZSEtP** (g), and **ZSPhP** (h) at 180 °C.

Table S1. Formulas of layered phosphonates prepared together with results of elemental and thermogravimetric analyses. Values in the first row are experimentally found, values in parenthesis are calculated.

host	formula	Elemental analysis % found(calc)				Weight loss %	
		C	H	N	S	first	total
Guanidine hydrochloride, room temperature							
α-ZP	two phases	-	-	-	-	-	-
γ-ZP	host	-	-	-	-	-	-
ZSEtP	ZSEtP·1.1Gua·0.5H_2O	10.44 (11.31)	2.93 (3.07)	8.53 (8.54)	11.29 (11.85)	2.0 (1.66)	51.0 (50.22)
ZSPhP	ZSPhP·1.05Gua·0.5H_2O	21.44 (25.34)	2.37 (2.65)	7.06 (7.13)	8.10 (9.33)	1.5 (1.46)	57.2 (57.13)
Guanidine carbonate, room temperature							
α-ZP	ZP·1.25Gua·H_2O	4.18 (4.00)	2.92 (2.76)	13.85 (14.01)	-	6.8 (4.80)	32.0 (29.30)
γ-ZP	ZP·0.9Gua·H_2O	3.12 (3.05)	2.18 (2.42)	9.61 (10.67)	-	4.5 (5.08)	30.0 (25.17)
ZSEtP	ZSEtP·0.85 Gua·H_2O	10.52 (10.88)	2.58 (3.06)	6.64 (6.67)	11.59 (11.97)	6 (3.36)	51 (50.49)
ZSPhP	ZrSPhP·0.95Gua·H_2O	24.09 (25.02)	2.32 (2.72)	6.38 (6.42)	9.82 (9.28)	3.1 (2.90)	57.5 (57.34)
Guanidine hydrochloride, 180°C							
α-ZP	ZP·1.83NH_3·H_2O	0.44 (0.00)	2.69 (2.88)	7.58 (7.71)	-	5.8 (5.42)	20.6 (20.22)
γ-ZP	ZP·0.5Gua·H_2O	1.90 (1.82)	1.55 (1.98)	5.83 (6.35)	-	4.9 (5.45)	20.5 (19.82)
ZSEtP	ZSEtP·NH_3·H_2O	9.67 (9.56)	2.85 (3.00)	2.93 (2.79)	12.05 (12.76)	4.0 (3.59)	48.3 (47.22)
ZSPhP	two phases	-	-	-	-	-	-
Guanidine carbonate, 180°C							
α-ZP	ZP·1.75NH_3·H_2O	0.34 (0.00)	2.62 (2.82)	7.40 (7.41)	-	5.5 (5.44)	20.2 (19.81)
γ-ZP	ZP·1.75NH_3	0.22 (0.00)	2.71 (2.34)	7.76 (7.83)	-	0.5 (0.00)	15.8 (15.28)
ZSEtP	amorphous	-	-	-	-	-	-
ZSPhP	amorphous	-	-	-	-	-	-

α-Gua

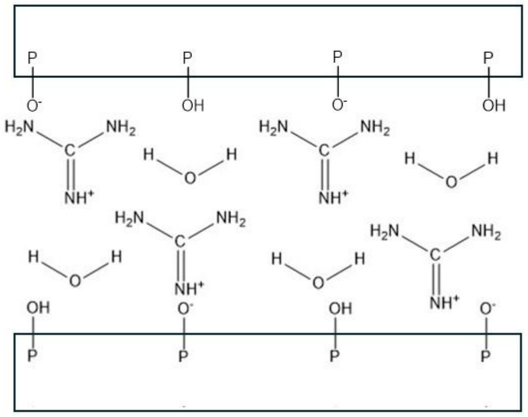

γ-Gua

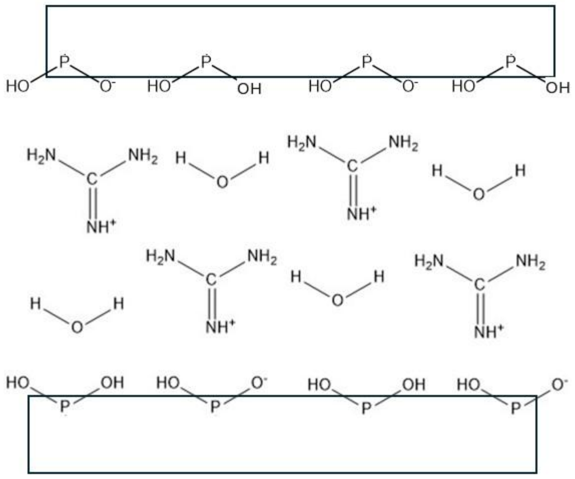

ZSEtP-Gua

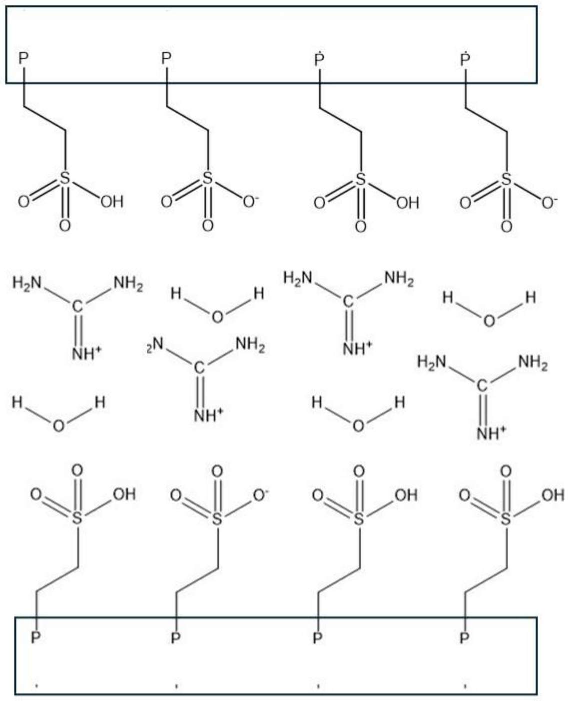

ZSPhP-Gua

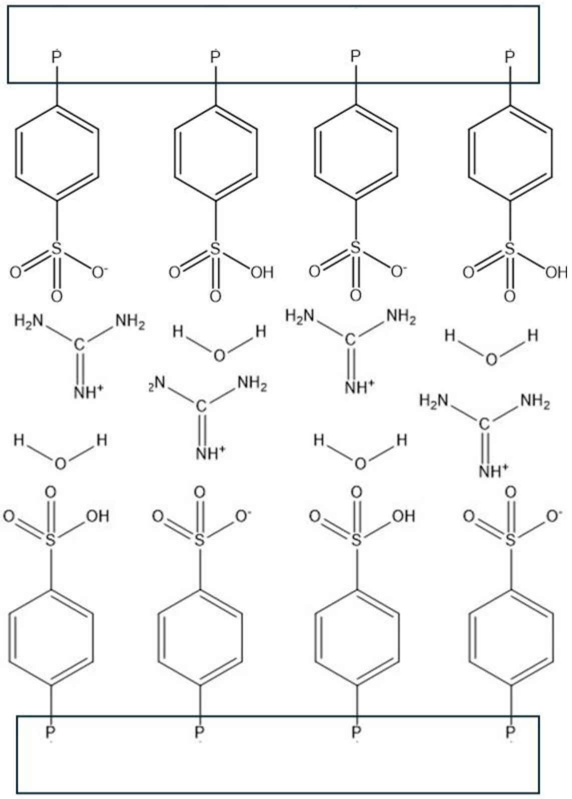

Scheme S1. Schematic representation of the intercalates prepared.

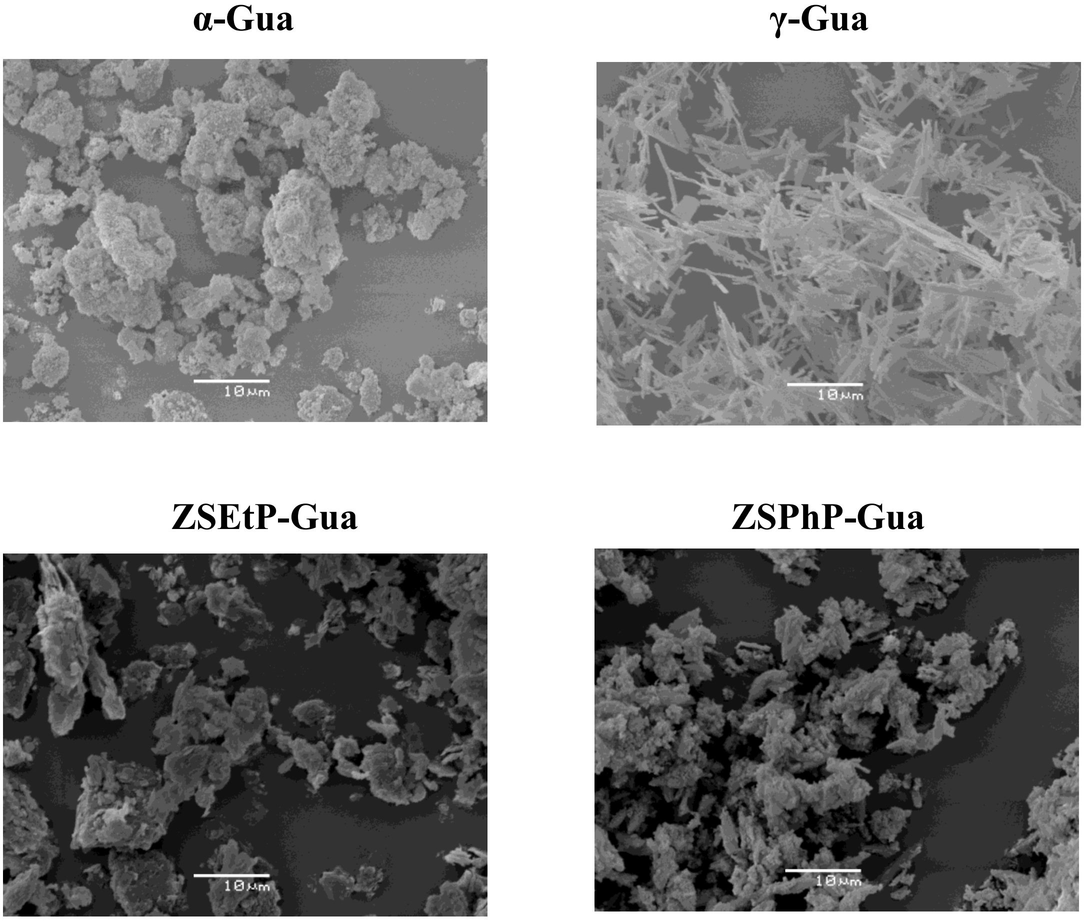

Figure S3. SEM images of guanidine intercalates.

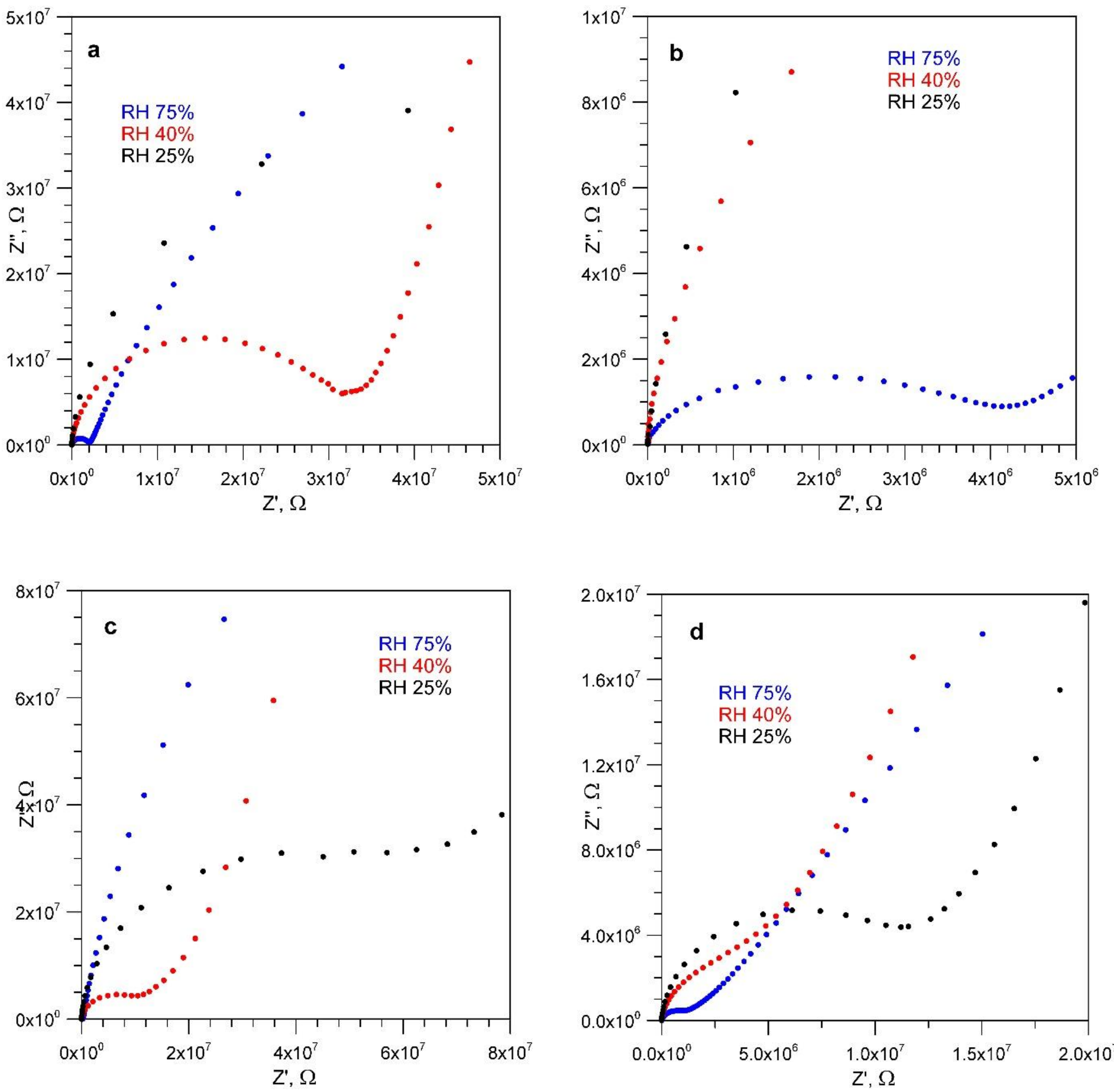

Figure S4. Complex impedance plots for **α-Gua** (a), **γ-Gua** (b), **ZSEtP-Gua** (c), and **ZSPhP-Gua** at 25 °C with variation of RH.

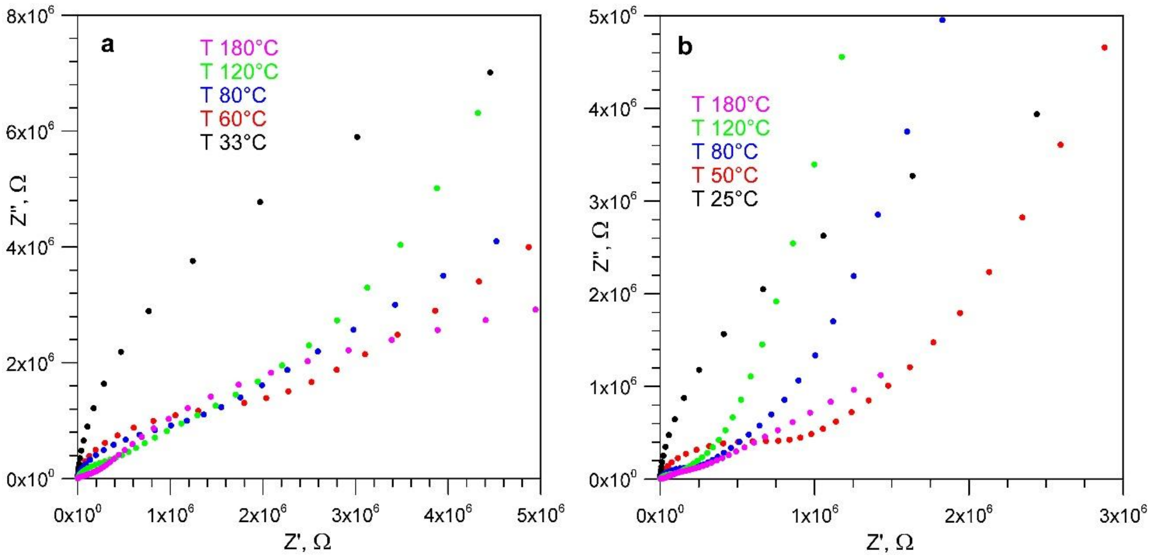

Figure S5. Complex impedance plots for guanidine intercalated **ZSEtP-Gua** (a) and **ZSPhP-Gua** (b) at various temperatures.

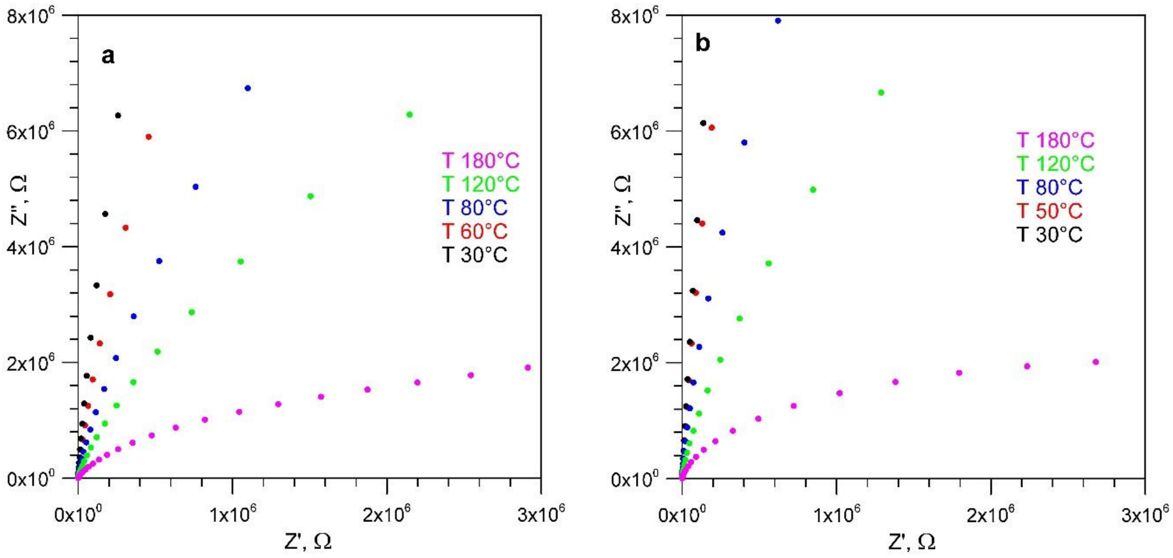

Figure S6. Complex impedance plots for dehydrated **ZSEtP-Gua** (a) and **ZSPhP-Gua** (b) at various temperatures.

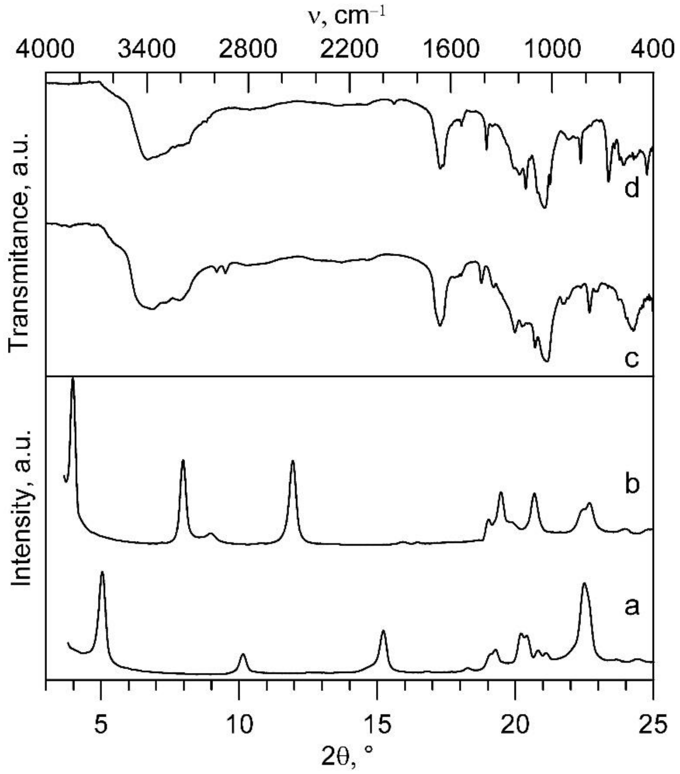

Figure S7. Powder X-ray diffraction pattern of **ZSEtP-Gua** (a) and **ZSPhP-Gua (b)** and IR spectra of **ZSEtP-Gua** (c) and **ZSPhP-Gua** (d) after conductivity measurement and rehydration.
